# 3-D structure of extracellular matrix regulates gene expression in cultured hepatic stellate cells to induce process elongation

**DOI:** 10.1186/1476-5926-2-S1-S4

**Published:** 2004-01-14

**Authors:** Mitsuru Sato, Takeya Sato, Naosuke Kojima, Katsuyuki Imai, Nobuyo Higashi, Da-Ren Wang, Haruki Senoo

**Affiliations:** 1 Department of Anatomy, Akita University School of Medicine, Akita 010-8543, Japan

## Abstract

HSCs showed myofibroblast-like shapes when cultured on polystyrene surface or on type I collagen-coated surface, whereas HSCs cultured on type I collagen gel were induced to elongate cellular processes, suggesting that HSCs recognize 3-D structure of extracellular type I collagen fibrils and change their morphology and function. In this study we examined the differentially regulated gene expression by extracellular matrix (ECM) components by PCR-differential display (PCR-DD) analysis followed by cloning and FASTA homology search, and identified the mRNA species as a transcription factor SP1, breast cancer resistant protein (BCRP), dystonin, and KAP3B. Regulation of dystonin and KAP3B expression was confirmed by RT-PCR analysis. Thus, cell surface-binding to extracellular interstitial collagen may trigger intracellular signaling and alteration in gene expression, and HSCs not only produce various ECM components but also change their morphology and gene expression in response to ECM components adhering to the cells.

## Introduction

Hepatic stellate cells (HSCs) located between endothelial cells and parenchymal cells have been found to extend long processes and surround hepatic sinusoids *in vivo *[1]. HSCs are, however, altered to myofibroblast-like phenotype after isolation and repeated subculture using ordinary polystyrene culture dishes [2,3]. We have shown that HSCs cultured using type I collagen gel as a substratum exhibit an *in vivo *morphology with long cellular processes [3-5], suggesting the regulation of HSC morphology and function by extracellular matrix (ECM) components in the perisinusoidal space of Disse. However, little is known about what function is regulated by ECM components in HSCs.

Techniques based on PCR-DD analysis are useful for comparing differences in gene expression between cell populations. In this study we used PCR-DD approaches to identify and clone differentially expressed genes regulated by ECM components in cultured HSCs.

## Methods

HSCs were isolated and subcultured in DMEM containing 10% FBS. To examine the effects of ECM components on cell morphology and gene expression, HSCs were cultured on polystyrene surface, on or in type I collagen gel, and on Matrigel. For RT-PCR and PCR-DD analyses, total RNA was isolated from each HSC culture, reverse transcribed. For PCR-DD analysis, the 1st strand cDNA was amplified by PCR using arbitrary primer sets (Clontech) in the presence of [alpha-^33^P]dATP. The PCR products derived from differentially expressed mRNAs were identified after electrophoresis and autoradiography, and the mRNA species were verified by cloning, sequencing, and FASTA data base search. Expression of the identified mRNAs and the regulation by ECM were confirmed by RT-PCR analysis.

## Results and Discussion

**Figure 1:**
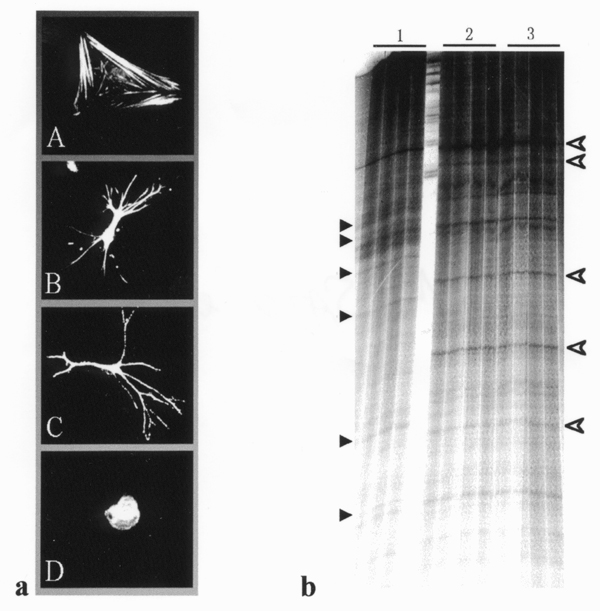
Alteration in cell shape (a) and gene expression (b) by ECM in cultured HSCs. **Fig. 1a **shows HSC shape after culturing on polystyrene surface (A), on type I collagen gel (B), in type I collagen gel (C), or on Matrigel (D). **Fig. 1b **indicates a representative PCR-DD showing differentially expressed transcripts in HSCs cultured on polystyrene (1), on type I collagen gel (2), or in type I collagen gel (3). Black and open arrowheads indicate the PCR products derived from down- and up-regulated transcripts, respectively, by extracellular type I collagen.

HSCs showed flattened, myofibroblast-like cell shapes with well-developed stress fibers when cultured on type I collagen-coated surface, as well as on polystyrene surface, whereas rounded shapes without cell spreading and process elongation when cultured on Matrigel (Fig. 1a) [3-5]. However, HSCs cultured using type I collagen gel as a substratum induced to elongate long cellular processes and exhibited asteroid shapes (Fig. 1a) [3-5], suggesting that HSCs can recognize two- or three-dimensional structure of extracellular type I collagen fibrils and change their morphology and function. We have reported the involvement of intracellular signaling events including protein kinases, PI-3 kinase, small G-proteins, microtubule-associated protein (MAP2) and cytoskeleton reorganization in process elongation in HSCs cultured using type I collagen gel. The results from PCR-DD analysis (Fig. 1b) revealed that several mRNA species were differentially regulated by extracellular type I collagen used as substratum in cultured HSCs. The PCR products were reamplified, cloned and then sequenced, as followed by FASTA homology search. Among the identified mRNAs a transcription factor SP1, breast cancer resistant protein (BCRP), dystonin, and KAP3B were found to be differentially regulated by extracellular type I collagen. SP1 has been reported to be engaged in the regulation of interstitial collagen gene expression, whereas dystonin and KAP3B are known to be involved in cytoskeleton, F-actin, intermediate filament, or microtubule function and/or assembly, suggesting a crucial role of these proteins in morphological and functional change in cultured HSCs. Expression in cultured HSCs and the regulation of dystonin and KAP3 by extracellular type I collagen was verified by RT-PCR analysis using the specific primer pairs (data not shown).

Taken together, cell surface binding to extracellular interstitial collagen fibrils may trigger intracellular signaling and alteration in expression of ECM components and cytoskeleton-related proteins such as MAP2, dystonin, and KAP3B, and finally induced cytoskeleton microtubule reorganization for process elongation. Therefore, HSCs not only produce various ECM components but also change their morphology and gene expression according to the ECM components adhering to the cells.
